# Supplementary material for: Fixation-pattern similarity analysis reveals adaptive changes in face-viewing strategies following aversive learning
Source: eLife. 2019 Oct 22;8:e44111. doi: 10.7554/eLife.44111 (PMC6805121; doi:10.7554/eLife.44111)
Supplement: Supplementary file 1. — (A) Mixed-effects modeling of the similarity matrices during the baseline phase with the Perceptual model shown in Figure 1B. (B) Mixed-effects modeling of the similarity matrices during the generalization phase with the Perceptual model shown in Figure 1B. (C) Mixed-effects modeling of the similarity matrices during the baseline phase with the Adversity Gradient model shown in Figure 1D. (D) Mixed-effects modeling of the similarity matrices during the generalization phase with the Adversity Gradient model shown in Figure 1D. (E) Mixed-effects modeling of the similarity matrices during the baseline phase with the CS+ Attraction model shown in Figure 1E. (F) Mixed-effects modeling of the similarity matrices during the generalization phase with the CS+ Attraction model shown in Figure 1E. [file elife-44111-supp1.docx]

# Supplementary File 1

### Supplementary file 1A

Mixed-effects modeling of the similarity matrices during **the baseline phase** **with** **the Perceptual model** shown in Figure 1B.

**Linear mixed-effects model fit by ML**

**Model information:**

**Number of observations 2072**

**Fixed effects coefficients 2**

**Random effects coefficients 148**

**Covariance parameters 4**

**Formula:**

**FPSA_B ~ 1 + circle + (1 + circle | subject)**

**Model fit statistics:**

**AIC BIC LogLikelihood Deviance**

**-278.51 -244.69 145.25 -290.51**

**Fixed effects coefficients (95% CIs):**

**Name Estimate SE tStat DF pValue Lower Upper**

**'(Intercept)' -0.12597 0.0052145 -24.158 2070 8.4562e-114 -0.1362 -0.11575**

**'circle' 0.093529 0.011633 8.0397 2070 1.4996e-15 0.070714 0.11634**

**Random effects covariance parameters (95% CIs):**

**Group: subject (74 Levels)**

**Name1 Name2 Type Estimate Lower Upper**

**'(Intercept)' '(Intercept)' 'std' 0.01266 0.0056794 0.028219**

**'circle' '(Intercept)' 'corr' 1 NaN NaN**

**'circle' 'circle' 'std' 0.075457 0.056786 0.10027**

**Group: Error**

**Name Estimate Lower Upper**

**'Res Std' 0.22222 0.21544 0.22922**

### Supplementary file 1B

Mixed-effects modeling of the similarity matrices during **the** **generalization phase with** **the Perceptual Expansion model** shown in Figure 1C.

**Linear mixed-effects model fit by ML**

**Model information:**

**Number of observations 2072**

**Fixed effects coefficients 2**

**Random effects coefficients 148**

**Covariance parameters 4**

**Formula:**

**FPSA_G ~ 1 + circle + (1 + circle | subject)**

**Model fit statistics:**

**AIC BIC LogLikelihood Deviance**

**-1731.2 -1697.4 871.61 -1743.2**

**Fixed effects coefficients (95% CIs):**

**Name Estimate SE tStat DF pValue Lower Upper**

**'(Intercept)' -0.11904 0.0040333 -29.515 2070 4.18e-160 -0.12695 -0.11113**

**'circle' 0.13803 0.014025 9.842 2070 2.2788e-22 0.11053 0.16554**

**Random effects covariance parameters (95% CIs):**

**Group: subject (74 Levels)**

**Name1 Name2 Type Estimate Lower Upper**

**'(Intercept)' '(Intercept)' 'std' 0.017918 0.01187 0.027046**

**'circle' '(Intercept)' 'corr' 1 NaN NaN**

**'circle' 'circle' 'std' 0.11179 0.092652 0.13487**

**Group: Error**

**Name Estimate Lower Upper**

**'Res Std' 0.15343 0.14874 0.15826**

### Supplementary file 1C

Mixed-effects modeling of the similarity matrices during **the baseline phase with the Adversity Gradient model** shown in Figure 1D.

**Linear mixed-effects model fit by ML**

**Model information:**

**Number of observations 2072**

**Fixed effects coefficients 3**

**Random effects coefficients 222**

**Covariance parameters 7**

**Formula:**

**FPSA_B ~ 1 + specific + unspecific + (1 + specific + unspecific | subject)**

**Model fit statistics:**

**AIC BIC LogLikelihood Deviance**

**-363.3 -306.94 191.65 -383.3**

**Fixed effects coefficients (95% CIs):**

**Name Estimate SE tStat DF pValue Lower Upper**

**'(Intercept)' -0.12597 0.0050119 -25.135 2069 6.8848e-122 -0.1358 -0.11615**

**'specific' 0.08272 0.017916 4.6171 2069 4.1301e-06 0.047585 0.11785**

**'unspecific' 0.10434 0.016232 6.4278 2069 1.6021e-10 0.072504 0.13617**

**Random effects covariance parameters (95% CIs):**

**Group: subject (74 Levels)**

**Name1 Name2 Type Estimate Lower Upper**

**'(Intercept)' '(Intercept)' 'std' 0.013135 0.006427 0.026843**

**'specific' '(Intercept)' 'corr' 0.74446 0.73891 0.7499**

**'unspecific' '(Intercept)' 'corr' 0.53826 NaN NaN**

**'specific' 'specific' 'std' 0.12886 0.10263 0.1618**

**'unspecific' 'specific' 'corr' -0.16199 NaN NaN**

**'unspecific' 'unspecific' 'std' 0.11113 0.086521 0.14275**

**Group: Error**

**Name Estimate Lower Upper**

**'Res Std' 0.21206 0.20546 0.21886**

### Supplementary file 1D

Mixed-effects modeling of the similarity matrices during **the generalization phase with the Adversity Gradient** model shown in Figure 1D.

**Linear mixed-effects model fit by ML**

**Model information:**

**Number of observations 2072**

**Fixed effects coefficients 3**

**Random effects coefficients 222**

**Covariance parameters 7**

**Formula:**

**FPSA_G ~ 1 + specific + unspecific + (1 + specific + unspecific | subject)**

**Model fit statistics:**

**AIC BIC LogLikelihood Deviance**

**-2013.8 -1957.5 1016.9 -2033.8**

**Fixed effects coefficients (95% CIs):**

**Name Estimate SE tStat DF pValue Lower Upper**

**'(Intercept)' -0.11904 0.0037523 -31.725 2069 2.4755e-180 -0.1264 -0.11168**

**'specific' 0.16048 0.018993 8.4494 2069 5.4388e-17 0.12323 0.19773**

**'unspecific' 0.11558 0.017208 6.7171 2069 2.3879e-11 0.081839 0.14933**

**Random effects covariance parameters (95% CIs):**

**Group: subject (74 Levels)**

**Name1 Name2 Type Estimate Lower Upper**

**'(Intercept)' '(Intercept)' 'std' 0.018228 0.012848 0.025862**

**'specific' '(Intercept)' 'corr' 0.82909 NaN NaN**

**'unspecific' '(Intercept)' 'corr' 0.72274 0.72118 0.7243**

**'specific' 'specific' 'std' 0.15391 0.12901 0.18361**

**'unspecific' 'specific' 'corr' 0.2128 0.20509 0.22048**

**'unspecific' 'unspecific' 'std' 0.13749 0.1147 0.16481**

**Group: Error**

**Name Estimate Lower Upper**

'Res Std' 0.13757 0.13329 0.14198

### Supplementary file 1E

Mixed-effects modeling of the similarity matrices during **the baseline phase with the CS+ Attraction model** shown in Figure 1E.

**Linear mixed-effects model fit by ML**

**Model information:**

**Number of observations 2072**

**Fixed effects coefficients 4**

**Random effects coefficients 296**

**Covariance parameters 11**

**Formula:**

**FPSA_B ~ 1 + specific + unspecific + Gaussian + (1 + specific + unspecific + Gaussian | subject)**

**Model fit statistics:**

**AIC BIC LogLikelihood Deviance**

**-354.79 -270.25 192.4 -384.79**

**Fixed effects coefficients (95% CIs):**

**Name Estimate SE tStat DF pValue Lower Upper**

**'(Intercept)' -0.12129 0.027412 -4.4246 2068 1.0164e-05 -0.17504 -0.067528**

**'specific' 0.085505 0.025383 3.3687 2068 0.00076919 0.035727 0.13528**

**'unspecific' 0.10422 0.016272 6.4048 2068 1.8583e-10 0.072307 0.13613**

**'Gaussian' -0.0093972 0.053725 -0.17491 2068 0.86116 -0.11476 0.095963**

**Random effects covariance parameters (95% CIs):**

**Group: subject (74 Levels)**

**Name1 Name2 Type Estimate Lower Upper**

**'(Intercept)' '(Intercept)' 'std' 0.04337 0.020094 0.093607**

**'specific' '(Intercept)' 'corr' 0.99498 NaN NaN**

**'unspecific' '(Intercept)' 'corr' -0.090915 NaN NaN**

**'Gaussian' '(Intercept)' 'corr' -0.97187 NaN NaN**

**'specific' 'specific' 'std' 0.14892 0.11712 0.18935**

**'unspecific' 'specific' 'corr' -0.19016 NaN NaN**

**'Gaussian' 'specific' 'corr' -0.99057 -0.9907 -0.99044**

**'unspecific' 'unspecific' 'std' 0.11145 0.08658 0.14345**

**'Gaussian' 'unspecific' 'corr' 0.32289 NaN NaN**

**'Gaussian' 'Gaussian' 'std' 0.067821 0.025734 0.17874**

**Group: Error**

**Name Estimate Lower Upper**

'Res Std' 0.21194 0.20535 0.21874

### Supplementary file 1F

Mixed-effects modeling of the similarity matrices during **the generalization phase with the CS+ Attraction model** shown in Figure 1E.

**Linear mixed-effects model fit by ML**

**Model information:**

**Number of observations 2072**

**Fixed effects coefficients 4**

**Random effects coefficients 296**

**Covariance parameters 11**

**Formula:**

**FPSA_G ~ 1 + specific + unspecific + Gaussian + (1 + specific + unspecific + Gaussian | subject)**

**Model fit statistics:**

**AIC BIC LogLikelihood Deviance**

**-2008.2 -1923.6 1019.1 -2038.2**

**Fixed effects coefficients (95% CIs):**

**Name Estimate SE tStat DF pValue Lower Upper**

**'(Intercept)' -0.1272 0.018548 -6.8577 2068 9.2098e-12 -0.16357 -0.090822**

**'specific' 0.15564 0.023446 6.6381 2068 4.0482e-11 0.10966 0.20162**

**'unspecific' 0.11579 0.017154 6.7502 2068 1.9111e-11 0.082152 0.14943**

**'Gaussian' 0.016341 0.035432 0.4612 2068 0.6447 -0.053144 0.085827**

**Random effects covariance parameters (95% CIs):**

**Group: subject (74 Levels)**

**Name1 Name2 Type Estimate Lower Upper**

**'(Intercept)' '(Intercept)' 'std' 0.053691 0.029469 0.097824**

**'specific' '(Intercept)' 'corr' 0.89896 NaN NaN**

**'unspecific' '(Intercept)' 'corr' 0.65757 NaN NaN**

**'Gaussian' '(Intercept)' 'corr' -0.99905 -0.99907 -0.99903**

**'specific' 'specific' 'std' 0.1731 0.14336 0.20902**

**'unspecific' 'specific' 'corr' 0.26112 NaN NaN**

**'Gaussian' 'specific' 'corr' -0.91722 NaN NaN**

**'unspecific' 'unspecific' 'std' 0.13697 0.11428 0.16417**

**'Gaussian' 'unspecific' 'corr' -0.62407 -0.62477 -0.62336**

**'Gaussian' 'Gaussian' 'std' 0.071235 0.029237 0.17356**

**Group: Error**

**Name Estimate Lower Upper**

**'Res Std' 0.13739 0.13312 0.1418**
